# A Practical Treatise on Midwifery

**Published:** 1845-04

**Authors:** 


### Art. VII.

*A Practical Treatise on Midwifery.* By M. Chailly, Doctor of Medicine, Professor of Midwifery, &c. &c. Illustrated with 216 Woodcuts. *A work adopted by the Royal Council of Public Instruction.* Translated from the French and Edited by Gunning S. Bedford, a.m. m.d., Professor of Midwifery and the Diseases of Women and Children in the University of New York.—*New York,* 1844. 8vo, pp. 530.

2. *A Practical Treatise on Midwifery; exhibiting the present advanced state of the Science.* By F. J. Moreau, Professor of Midwifery, &c. &c. Translated from the French by Thomas Forrest Betton; and edited by Paul B. Goddard, a.m. m.d., Lecturer on Anatomy, &c. &c. With Eighty Plates, comprising numerous separate illustrations.—*Philadelphia,* 1844. 4to, pp. 236.

The activity of the American press in transferring to its own literature the best works of foreigners, as well by simple republication, as by the laborious process of translation, is universally known. We have a striking instance of this in the two volumes before us—two elaborate treatises on the same subject, translated by American physicians, and published almost simultaneously at the two great rival cities of the United States. We shall notice them separately, and in the order in which they reached us.

I. In our Journal for October 1842, we have already briefly considered M. Chailly's original work, and expressed our favorable opinion of its minute details regarding the steps into his own operation, and by describing the particular forms of instruments which he has found best adapted for his proceedings.

The preparation of cleft palate, a dissection of the parts in the usual condition of the throat, a variety of diagrams, instruments, &c. were on the table, to illustrate the views of the author." (Medical Times, Dec. 21, 1844.)

merits. Our notice of the translation will not, therefore, be much extended but we may advert to some statements and opinions of M. Chailly, which we have not previously commented upon, as well as to some of the brief additions made by the translator in the form of foot-notes.

In cases of procidentia of the funis, M. Chailly says, that when the orifice is completely dilated, and the contractions have attained a certain intensity, the accoucheur should introduce two or three fingers, and even the whole hand, if the condition of the external parts will permit, in order to push up the cord through the membranes: then profiting by an energetic contraction, he will rupture the amniotic sac, either with the hand introduced, or by means of an ordinary pen, carried along the hand supporting the cord, and pushed forward with the other: at the moment the fluid escapes, the head places itself exactly over the uterine orifice, while the cord, sustained by the extremity of the fingers, is carried above the superior strait, and is protected from compression. That this practice will always fail we do not assert or believe; but we do assert and believe that it will fail in the great majority of cases. The practice recommended by Dr. Michaelis of Kiel,* is, we think, more to be depended upon, and safer for the mother in the hands of average practitioners. It seldom happens that the prolapsus of the funis is discovered before the membranes are ruptured.

M. Chailly is one of the very few French writers who admit the propriety of opening the head of the living child, when the nature of the case would, without this expedient, expose the mother to great danger and perhaps destruction. We have never, for a moment, doubted the propriety and perfect morality of the principle which would guide the English accoucheur in such melancholy instances; and for the sake of suffering women, we are happy to find M. Chailly's influence exerted to remove the prejudices of his countrymen upon so important a point. He very wisely lays it down as a rule, that when we are driven to the distressing alternative of opening the head of the child to save the life of the mother, the opinion of several practitioners should be taken. This is desirable for the security of our own reputations, as well as due to the fears and feelings of the mother and her friends.

Perhaps there is no point in the practice of midwifery that has been more warmly discussed and less definitely settled than the exact degree of pelvic contraction that prevents us from extracting the child through the natural passages. M. Chailly is not very precise upon this point.

"*The pelvis less than two inches.* In this case, we cannot think of extracting the child through the natural passages: for supposing even that the instrument could be introduced, it would then not always be possible to remove the child; and this removal would require immense efforts, which lacerate and bruise the organs of the mother, and expose her to nearly the same danger as the Cæsarean section. In this event, one of the two individuals would be sacrificed, and the other would have a very slight chance of life; while the Cæsarean operation saves (may save?) the life of the child, and leaves the mother nearly as many chances of safety as cephalotripsy." (p. 294.)

The instrument referred to by M. Chailly is the cephalotribe; but his arguments will apply with equal force to any of the instruments employed in this country for the same purpose, where there is only just room to re-

* British and Foreign Medical Review, vol. I, p. 588.

move the child by the natural passages, even if the practitioner is very skilful in their employment. Practical experience, and that alone, can convey to the mind a correct notion of the difficulties to the operator, and of the suffering and danger of the mother in such instances. Burns and others have offered very sensible remarks upon this subject. Dr. Burns very truly says that it ought not to be forgotten that it is one thing to extract, and another to extract safely, in extreme deformity. It is possible, after much exertion, to bring away the child, but every one must have seen the mother lost, in cases where the capacity of the pelvis was far from being reduced to the minimum. Sometimes the uterus is ruptured, sometimes the soft parts slough, but oftener the patient dies, either of peritonitis, or the belly swells without pain, and she sinks. We ought to be satisfied, not only that we can bring through the child, but that we can do so without so much violence as must, in all probability, kill the mother. "I question much if extreme cases be not as dangerous to the patient as the Cæsarean operation; certainly they are more painful."* We have seen three cases in which the child was removed by the crotchet, forceps, &c., in consequence of great pelvic contraction, all of which ended fatally. The operator was dexterous and patient; the suffering of the women during the operation was sickening to behold.

In discussing the mode of employing the forceps, M. Chailly asks, "towards what part of the pelvis should the branches be directed? Should they be carried directly to the place they are intended ultimately to occupy? or should they be directed first to the posterior part of the pelvis to be afterwards brought to their proper situation by the hand, introduced into the maternal organs, and by a vibratory movement adapted to the handle of the instrument? I adopt this latter mode after the example of M. P. Dubois." (p. 311.) A very good practical authority, but here, we think, a fallible one. The translator "can see no advantage in this rule; but, on the contrary, trouble may arise from it. Suppose, for example, the head is in the pelvic cavity, and it becomes necessary to apply the forceps. If the head be situated directly in the pelvis, with either the occiput or forehead regarding the pubes, nothing would be easier than to introduce the instrument, taking the hand previously passed into the vagina as a guide, on the sides of the head at once, without previously carrying the blades to the posterior part of the pelvis. This latter procedure might result in injury to the scalp of the child, in consequence of the efforts made to bring the blades to the lateral portions of the pelvis. But, if carried to these points when first introduced, no such difficulty would follow." (Foot-note, p. 311.) Here it appears to us, that a comparatively trifling and not very probable objection to the mode of practice advocated by M. Chailly is adduced, while a much greater and more likely mischief from it is passed over, namely, injury to the soft parts of the mother by this "vibratory movement" of the forceps, which, as a general rule, ought to be and may be avoided by applying the blades of the instrument to the sides of the head of the child at once, if the mother is placed in a proper situation. We quite agree with the translator as to the impropriety, we would even say folly, of using the forceps secretly; the opinion we formerly† gave upon this point, is almost literally that which is embraced

* Burns's Midwifery, 8th edit. p. 468.

† British and Foreign Medical Review, October 1842, p. 372.

in Dr. Bedford's note at p. 315. Dr. Bedford might very safely have spared the long critical note at p. 316 upon Dr. Ramsbotham's opinions as to the use of the forceps. He is quite mistaken in supposing that Dr. Ramsbotham would "do nothing, because the ear of the child cannot be felt," as is clear from what he says upon the subject.* We object to the advice given by M. Chailly, of introducing the whole of the hand, except the thumb, into the maternal organs when we employ the forceps. As a very general rule, the introduction of one or two fingers between the head of the child and the soft parts of the mother is quite sufficient to guide the blade of the instrument safely. Again, we must be permitted to say that no assistant is necessary to hold the branch of the forceps that is first applied in its situation. He who possesses but even moderate dexterity, will be able very easily to keep the blade in its position by his finger and thumb, while he introduces the second blade. M. Chailly advises us, when we have applied the forceps, to "bind the handles strongly together by a napkin." This expedient is generally unnecessary, and often very injurious to the child, for the reason properly pointed out by the translator, that it keeps up a constant, severe, and sometimes fatal pressure upon the head. We can make sufficient pressure with our hand, and this power we can, of course, increase or diminish from time to time, according to the exigencies of the case we have to manage.

Upon another point, too, we agree with Dr. Bedford, and differ entirely from M. Chailly. The latter would perform the operation of turning without the consent of the patient, and would "carefully conceal from her the true nature of it." (p. 350.)

Dr. Bedford's advice is surely more judicious; first, that the necessity of interference should be impressed upon the patient's mind; and secondly, that what we are about to do should be frankly communicated to her. If the practitioner is kind in his manner, and judicious in his mode of thus acting sincerely with his patient, we are quite sure that ninety-nine women in a hundred will bear the pain of the operation with much more calmness and resignation than they would if they were kept in utter ignorance of what was doing or about to be done. M. Chailly, in common, as we thought with all other accoucheurs, would *not* grease the palm of the hand preparatory to turning, that he might have a firmer hold of the fetal limbs when he had grasped them : Dr. Bedford *would* do so, from the, in our opinion, imaginary fear, that the dry palm of the hand might come in contact with and injure the organs of the mother. The various steps in the operation of turning are rendered very clear to the student by the different woodcuts.

The cephalotribe, or compressing forceps of the head, invented by Baudelocque, nephew of the celebrated accoucheur, is regarded by M. Chailly as "a most precious instrument, which cannot be too positively defended against its traducers." In this country little or nothing is practically known of this instrument, the object of which is to supersede the use of the perforator and crotchet. We can readily believe M. Chailly's assurance, that "the firmest and most thoroughly ossified heads would readily yield under the pressure of this instrument;" but the most important question is, can the cephalotribe be used with as much safety as the instru-

* Principles and Practice of Obstetric Medicine, by Dr. F. H. Ramsbotham, p. 329.

ment it is intended to discard? Mr. Cooke* tells us that there are eleven cases on record in which the cephalotribe has been used, and in all, he believes, with success. In all, too, the Cæsarean operation had been considered to be absolutely necessary from the impossibility which existed of delivering by the crotchet.†

In cases where we may be compelled to leave some portions of the after-birth in the uterus, M. Chailly very properly advises us to examine the patient from time to time, and ascertain whether these portions are detached; and if they are, they should be removed by the fingers. To guard against the danger of the absorption of these putrefied masses, recourse should be had to vaginal injections of a weak solution of chloride of lime. But injections into the womb itself, which have been often recommended, should be used with great caution. M. Housman and others have reported cases in which serious accidents were occasioned by the passage of the fluid into the abdominal cavity through the Fallopian tube. M. Chailly observed a similar fact at La Clinique, from an injection thrown into the womb: the patient experienced a sudden pain in the right iliac fossa: all the symptoms of local peritonitis manifested themselves at this point; but they were combated, and the patient recovered.

One more point of practical importance we must advert to, because but little attention is paid to it in this country, and it refers to cases in which the life of the patient may be at stake, and in which even a temporary arrest of the danger may be of the utmost importance: we allude to pressure on the abdominal aorta before its bifurcation, in cases of hemorrhage after delivery. In conjunction with other and well known means, M. Chailly advises as follows:

"While the accoucheur is making frictions on the hypogastrium, he should administer ʒj of ergot in a small quantity of water. At the same time, he directs the assistants to place cold compresses on the legs and thighs; and if the frictions should be insufficient to bring on uterine contraction, he must not wait for the action of the ergot, but immediately, with the extremity of the fingers of one hand, press on the abdominal aorta before its bifurcation; and he thus exerts with this hand, on which is superposed the other, a compression which, when well made, almost invariably arrests the hemorrhage.

"This means, which we owe to M. Baudelocque—nephew, and whether he originated it or not matters little—is one of the most precious discoveries with which the obstetric art has been enriched.

"M. d'Ornelas has quite recently cited in his thesis several cases of success obtained by this remedy.

"My father had recourse to it once with the happiest result; and in two instances I have also been enabled to preserve the lives of two women, who certainly would have died had it not been for this valuable agent.

"In one of these patients I made use of this compression for nearly two hours, when M. P. Dubois, whom I had sent for, arrived and witnessed the fact.

"The effect produced by this compression is so manifest, that the flow of blood is immediately checked; and if, by a movement of the patient, the aorta should escape from the hand, the hemorrhage recommences immediately.

"This compression, as is well understood, is not a curative remedy; but it permits us to gain time, a result the more important in an accident so fearfully rapid as uterine hemorrhage.

* London Medical Gazette, vol. xvii, p. 14.

† The details of these cases will be found in Baudelocque's Mémoire sur le Cephalotripsie, and in the No. for April 1835 of Journ. de Méd., by Lucas Championniere.

"Finally, while this compression is faithfully persevered in, and the flooding is thus checked, we must employ, with activity, the other means which are calculated to arrest it definitively. Cold water should be thrown into the rectum; two or three drachms of ergot, in two or three doses; the cold compresses to the legs and thighs should be constantly renewed; and an assistant should make continued frictions to the uterus.

"The compression of the aorta will be the more easy in proportion as the patient is less loaded with fatty matter; but, even when the female is very corpulent, it still may be practised with facility and successfully. In fine, immediately after the expulsion of the fetus, the uterus descends, and there is a space between the fundus of this organ and the intestines, which, for a period of nine months, having been pressed up towards the superior portion of the abdomen, do not immediately after the delivery resume the place they occupied before pregnancy. It is into this space that the hand passes with facility for the purpose of making the compression.

"It is sometimes extremely fatiguing to continue this compression, and we are occasionally obliged to be relieved by an assistant." (pp. 475-6.)

We quite agree with Dr. Bedford that ergot should never be considered among the *heroic* remedies in the treatment of uterine hemorrhage after the birth of the child. Some practitioners are in the habit of relying on it, as an all-sufficient agent in these cases; but they are in error, for if the hemorrhage is profuse, the patient may die before the ergot can act. It does not fall within our province to describe the detailed management of such cases; suffice it for us to say, that any internal medicines can be little if at all relied upon.

We here close our notice of M. Chailly's work, and of Dr. Bedford's additions as the editor. If it were only for the fact of this work containing the opinions of one, certainly, of the best obstetricians in France, namely, Paul Dubois, it would have strong claims to all those who are interested upon practical subjects of midwifery; for one great merit of the work is, that no idle speculations are indulged in; it is practical, and therefore useful and instructive. Dr. Bedford's translation reads smoothly and well, and is, upon the whole, unusually free from the Gallicisms that translators from French to English so commonly introduce.

II. In the exposition of the subjects of which he treats M. Moreau has adopted the following order. In the First Part of the work he gives an explanation of the organs which concur, in any manner, in the accomplishment of the various functions of reproduction, pertaining to women alone. These organs are described, in their physiological state, and in a state of rest; in their physiological state, and during the performance of their functions; in the anomalies which they present, whenever these anomalies can modify, oppose, or prevent the execution of the functions peculiar to these organs. The subject of generation is left to treatises on physiology. In this we conceive the author has acted wisely and in keeping with the title of his work. A "practical" treatise on midwifery can scarcely, with propriety, do more than contain a very superficial, and therefore unsatisfactory, sketch of so extensive, so difficult, and at present—notwithstanding all the time and talent that have been expended on it—so unsettled a department of scientific inquiry. The Second Part of the work includes the study of the functions of the various organs, the structure of which is given in the first part. Part the Third is especially devoted to the various terminations of pregnancy; that is, to labour: to the distinctions between labours, and the numerous varieties they offer, either as terminating by the efforts of nature alone, or as requiring the interposition of art. In the Fourth Part the anomalies of pregnancy are considered, as its development, product, location, and termination. The Fifth and last division embraces an enumeration of the natural phenomena which supervene in the woman after delivery, and in the child at birth.

Such are the limits—and such the arrangement of the text of the present work. And in order to render the treatise as useful as possible, very numerous plates are added, of which after an attentive and even studious inspection, we can scarcely speak too highly, whether we regard their execution, or the happiness with which the various subjects for graphic illustrations are selected.

As the introductory parts of the work present no field for originality of description, we shall confine our notice of the volume to various points of practical moment that we have noted down as worthy of comment, and which are more or less peculiar to the author.

*Retroversion.* M. Moreau is surprised that Boyer, of well known talent and experience, has stated that retroversion of the uterus scarcely ever occurs in the virgin state. "We have observed it much more frequently in the empty uterus than during pregnancy." (p. 55.) That this displacement of the uterus may occur, from disease enlarging it to a certain degree, and therefore in the unimpregnated condition, we do not deny; but we are very doubtful of the fact of its having been found to exist "more frequently" in the empty uterus, and our assent to the statement, or the removal of our doubts as to some fallacy in the observation and diagnosis that have led to it, could only have been obtained by a very satisfactory account of cases in point.

From the difficulty we have experienced in replacing a retroverted uterus by manual assistance alone, we can easily conceive the advantage of the plan, in some cases, which M. Moreau mentions, and which he has found the most successful. It consists in placing the patient on one side, and introducing a rod, eight or ten inches long, furnished at one end with a tampon of linen well greased, into the rectum, in order to push the fundus of the uterus from below upward, whilst, with two fingers in the vagina, the os uteri is drawn downward and backward. This method originated with Evrat more than thirty years ago, and was adopted in the following case. A woman in the fifth month of pregnancy had retroversion of the womb. Various practitioners, Coutouly among the number, tried in vain to reduce it. M. Evrat, in the presence of his colleagues, placed the woman on her left side, introduced into the rectum the instrument described, while with two fingers in the vagina he seized the os uteri, and then moving his fingers and the rod in contrary directions, he restored, but not without difficulty, the uterus to its normal position. Notwithstanding the exertions used to reduce the organ, pregnancy was not interrupted and the patient was happily delivered of twins. And here we may observe that abortion by no means so necessarily happens, when the uterus has been retroverted, and even when some difficulty has been experienced in its reposition, as writers in general would lead us to believe. In five cases* that occurred to Hesse of this displacement, the patients went their full time.

* Busch's Journal, Bd. 8, Heft i, p. 122.

*Inversion.* It has been often stated, that when that very serious accident inversion of the uterus has taken place, nature, from the influence of some accidental circumstance, has been known to effect, spontaneously, a reduction, until then regarded as impossible. As the facts in support of this assertion are far from numerous we give the following case from M. Moreau. De la Berre, a surgeon, having retired to a chamber near that in which his wife was about to be delivered, was suddenly called to her aid after the birth of the child. The midwife had completely inverted the uterus in endeavouring to extract the placenta, and thinking it was a false conception, attempted to pull it away, with the assistance of another matron as ignorant as herself. Ineffectual attempts were made at reduction. Serious hemorrhage took place and continued. Six months afterwards in getting out of bed, the lady fell on the floor. She felt at the moment an extraordinary motion in her belly accompanied by very acute pain, and followed by syncope. On recovering she found that the tumour had disappeared: the inversion was reduced. The fact was communicated to the Royal Academy of Surgery. A similar case happened to Baudelocque several years after. Osiander* we find confirms this fact. "In some cases," he says, "nature alone effects reduction when art has failed. I know myself an instance of this kind."

*The modifications of the uterine and fetal circulation during labour* are not usually very satisfactorily touched upon by obstetrical writers. We give therefore M. Moreau's explanation of these changes.

"The first effects of the uterine contractions are to determine the shortening of the fibres, the diminution and contraction of the parietes, and the universal decrease of the cavity of the uterus: therefore, these changes cannot take place without producing others in the reciprocal relations of the constituent parts of the uterus and contained ovum. The uterine vessels, being compressed, decrease, their caliber diminishes, and, as they cannot undergo a retraction equal to that of the uterine fibres, their direction changes, their flexuosities increase, and the circulation through the parietes of the uterus is evidently modified, diminished, and slackened. The ovum, embraced on all sides by the uterine parietes, which contract and tend to expel it, at first resists, is then detached, and at last yields to the impulsion: but, during the struggle between the ovum and the expelling power, the membranes and the placenta, being extended, pressed outwardly by the uterus, inwardly by the fœtus and the amniotic fluid, experience changes which it is important to examine.

The placenta, diminished by a double pressure, can no longer receive the blood and the materials which it drew from the mother: if it still receives the blood which comes from the fœtus, it cannot transmit it to the uterus; hence arise numerous modifications in the fœtal circulation, which vary infinitely according to the slow or rapid, feeble or vigorous progress of the process of labour. During the contractions the uterus allows a smaller quantity of blood to pass in proportion to the energy and continuance and degree of shortening of its fibres: it therefore follows that if, in the first contractions, which are generally feeble, some fluids pass from the uterus to the placenta, a period occurs at which all communication between these organs ceases.

The placenta, no longer receiving any fluids from the uterus, cannot return to it the blood proceeding from the umbilical arteries: this blood, the residuum of fœtal nutrition, analogous to the venous blood of the adult, unfit for the support of life, is then obliged to return to the fœtus in the same state in which it left it, that is, without having undergone the alteration it should have experienced in the

* Die Ursachen und Hülfsanzeigen der unregelmässigen und schweren Geburten. Zweite Auflage, 1833, p. 145.

placenta. The contraction ceasing, the circulation again takes place as before the commencement of labour, and continues in the same manner during the whole time that the fœtus remains entire. But when the bag of waters is ruptured, and the amniotic fluid discharged, the contractions become more acute, longer, and closer together: the relations of the uterus and placenta change again; the former has diminished in proportion to the extent of dilatation of its orifice and the quantity of fluid discharged. The placenta, flacid, soft, not retractile, cannot follow the reduction of the uterus, and folds on itself: its harmonious relations are at an end: in the interval of the contractions, it can no longer send blood to the uterus: then the placental tissue becomes congested, the vessels are filled and distended, and soon can no longer receive the blood contained in the umbilical arteries: the latter are engorged, in their turn, and can no longer admit that conveyed to them by the divisions of the aorta: the aorta itself, not being emptied, can no longer receive the blood sent by the heart, and so on, there gradually supervenes an irregularity and perplexity in the general circulation, and a congestion of the whole sanguine vascular system of the fœtus. If this state continues, visceral congestion ensues, the vessels are ruptured, effusions take place under the pericranium, or are more deeply seated, and death is frequently the inevitable result. The fœtus is then born with a swollen, tumefied, and livid face, resembling a subject asphyxiated by carbonic acid gas: in short, perfectly apoplectic. If the contractions, on the contrary, have been very slow and not energetic, the fœtus, receiving no blood and constantly sending it out, is exhausted, and exhibits a condition of paleness, softness, flaccidity, and more or less complete anemia.

These phenomena and their causes being known, a host of therapeutic indications may be thence deduced, applicable either to our conduct during the progress of labour, or to the child at its birth. Thus, it may be easily seen that under certain circumstances it will be useful to endeavour to retard the progress of the labour, in others to accelerate it: that, in some cases, the preservation of the child will be owing to the promptness with which blood is taken from it, and that, in others, its life can be saved only by husbanding the little that remains." (p. 104.)

We cannot altogether agree with our author as to the extreme facility, with which he thinks, that "false pains" may always be distinguished from those owing to uterine contraction, by their "location and characters," and that we "cannot mistake" the one for the other, "however little attention we may give them." True, that an experienced practitioner will rarely fall into mistakes upon the subject, but it is also true that an examination per vaginam is not seldom required to lead to a correct diagnosis. Again, the following statement does not accord with our experience—now of more than thirty years—nor with the general opinion of accoucheurs. "We believe we may say that this sign,"—the *show*—previous to labour, "is frequently wanting, for it *is scarcely ever observed except in primiparous women*, or in cases of rapid labour, or when the fetus is very large: under opposite circumstances it rarely occurs." (p. 145.) To the latter part of this statement we gravely demur.

*Presentations.* M. Moreau claims for himself, the by no means unimportant merit, of being the first publicly to declare that cases of face presentation ought to be included among those which can terminate by the efforts of nature alone: "at least we have advanced this opinion long before the publication of the works of M. Velpeau and Madame La Chapelle." (p. 151.) Be this as it may, we must still, not from mere courtesy, give to Madame La Chapelle* the credit of having, more

* Prátique des Accouchemens. Par Madame la Chapelle, tome i, p. 367.

satisfactorily than other writers, described very explicitly the mechanism of face presentations, and also of refuting the erroneous views of many who had before taught that such cases were almost necessarily accompanied by great difficulty, if not danger to the *mother.* We are vexed to find M. Moreau accusing Denman of wishing to establish it as a rule of practice, that in presentations of the superior extremities, the "spontaneous evolution" of the fetus might be depended upon. Denman never committed so great and glaring a blunder, as we have elsewhere shown. Will M. Moreau permit us, with much respect, to caution him against quoting the opinions of writers at second-hand. We know that this allegation against Denman has been frequently made by English as well as foreign writers, but we know also, that it is unfounded. Denman regarded the spontaneous evolution of the fetus as an occurrence that might happen, but not as one that could be looked for, with any degree of confidence, or as one that could at all justify a departure from the well known rule of practice, in cases of *transverse* presentations.*

We have another protest to enter against M. Moreau. The following sentence excited a smile and some astonishment. Need we say that it has not a touch of one very essential quality—what that quality is politeness precludes us from formally stating. "In England, women generally refuse to allow any examination during labour, and the accoucheur is sent for to be, as it were, merely a spectator of a process left to nature." (p. 160.) The ladies of the United States are also included in this accusation of excessive folly. Are we to infer, from the silence of the translator and editor, American physicians, that the charge is well founded?

M. Moreau rarely uses the *ergot* of rye. He considers it injurious in certain circumstances, and "that its action is neither so constant nor so efficacious as many suppose." (p. 163.)

*Obliquity of the uterus in labour.* In speaking of the anterior obliquity of the uterus, a practical point concerning the management of which there is much diversity of opinion, M. Moreau says,

> "The woman is to be laid on her back, with a cushion under the breech, in order to establish the parallelism between the axis of the uterus and that of the superior strait: the fundus of the organ is pushed upward, a finger introduced into the vagina, in order, in the interval of the pains, to seek the os uteri, which is found on a level with the promontory of the sacrum, and to draw it gently forward." (p. 167.)

In our opinion the much safer direction, so far however as regards the manual interference alone, would be, to trust to time and patience in such cases. Dr. Robert Lee says,† in reference to the practice advised by M. Moreau and others, "this is one of the many dangerous manœuvres described by writers which ought never to be thought of." The fact we take it is this: that in the course of some time after the practitioner has been drawing forward the os uteri, sometimes gently, and sometimes, as we know, with reprehensible roughness, from an exaggerated apprehension of the danger of this anterior obliquity, he finds the os uteri in a more favorable position, and prides himself upon the benefit he

* Denman's Introduction to the Practice of Midwifery, 7th edition by Waller, p. 355.
† Lectures on the Theory and Practice of Midwifery, p. 245.

has conferred by his interposition, which is, however, in truth, to be referred to the gradual efforts of nature, which have remedied the malposition, as they almost always will, with time and patience, if the patient is placed on her back and the uterus is properly supported by a broad bandage applied round the body. We admit that there are some rare exceptions to this desirable termination, and examples are to be found in Baudelocque.*

*Hemorrhage.* Of the use and effects of plugging the vagina in appropriate cases of uterine hemorrhages M. Moreau thinks highly, but he also thinks that sometimes the flooding may be so copious and so sudden as to justify the plan advised by Celsus, namely, forcible dilatation of the os uteri. This must always be a very fearful expedient; we can hardly conceive it to be the lesser of two evils, so long as we can plug the vagina and restrain the hemorrhage. At all events it is not a plan to be recommended in print, or even thought of in practice, without the absolute necessity for instant delivery is clearly established.

*Turning.* We are scarcely satisfied with the observation that in performing the operation of *turning*, "the manœuvre is best performed during the absence of pains." (p. 180.) The safer direction would surely be that the attempt to turn *must* not be made during the continuance of pains.

M. Moreau considers the use of the *cephalotribe* as liable to more objections than cephalotomy.

*Œdema of the labia.* We should object to the free incisions advised by M. Moreau in cases of œdema of the labia pudendi, rendering delivery difficult. Small punctures will be sufficient to let out the contained fluid. Large ones would be somewhat dangerous, because parts that are distended by œdema have lost their tone, their disposition to recover from injury; and therefore, free incisions would be apt to run into the very mischief we wish to avoid, viz. gangrenous sloughs.

*Bulk of the child.* M. Moreau is inclined to believe that a debilitating regimen of the mother may have a decided influence over the offspring, and that it may be properly enforced in certain cases with the view of lessening the bulk of the child. We doubt the fact, and he confesses he once was opposed to it.

*Extraction of the placenta.* M. Moreau regards the practice of throwing injections into the unbilical vein of cold water, or mixed with vinegar or alcohol, with the view of destroying adhesions between the placenta and uterus, and of exciting the contractions of the latter, both as uncertain and not altogether safe. For example,

"The expulsion of the after-birth not taking place in a woman whose delivery had been perfectly natural, and traction on the cord having been ineffectually exerted, the physician in attendance injected some cold water into the umbilical vein. This injection produced no effect: it was repeated, but with vinegar and water. This time the woman almost immediately tasted the vinegar in her mouth; she was attacked with ptyalism and such an intense chill, that, during fifteen hours, it was with the greatest difficulty she could be kept warm. Nevertheless, as the after-birth was not expelled, we were sent for. We found the cord hanging in the vulva, and the placenta partly engaged in the os uteri, but not completely detached. We introduced our hand into the uterus, broke up the adhesions, and readily extracted it. All the symptoms ceased, the woman

* L'Art des Accouchemens, 7e ed., t. i, p. 166.

recovered, and has subsequently had several children. This curious fact seems to prove to us that injections of acidulated water into the umbilical vein are not so exempt from danger as might be at first supposed." (p. 218.)

At the end of the work M. Moreau gives an interesting paper on perforation of the perineum, and on the passage of the child through this part, which was read in 1830 at the Royal Academy of Medicine. As it is short we shall extract the case which gave origin to it:

"Madame D——, living in Paris, 19 or 20 years of age, pregnant for the first time, was delivered on the 3d of March 1815. Labour came on naturally, and followed a regular course; the child presented in the fourth position of the head; the os uteri dilated in five or six hours; the membranes were spontaneously ruptured. When the dilatation was complete the head descended without difficulty into the pelvic excavation; but, when on the point of escaping through the inferior strait, it had great difficulty in passing under the arch of the pubes. During a very acute pain, M. Evrat, thought that he felt the middle of the perineum corresponding to the palms of his hand, lose its thickness and elasticity, and yield sensibly to the pressure of the child's head. He was reflecting how he could prevent laceration of the perineum, which appeared to him on the eve of occurring, when a violent pain, the effect of which he could not oppose, expelled the child, but in such a way that the head, instead of escaping per vias naturales, passed through the perineum, leaving in front the posterior commissure of the vulva, and behind, the orifice of the anus, uninjured. The irregular wound resulting from this perforation extended to the right, in the direction of the ascending ramus of the ischium and descending of the pubes; anteriorly, beyond the posterior commissure of the vulva, and posteriorly, wound slightly around the anus; then it passed transversely from right to left, between the anus and vulva, nearly as far as the tuberosity of the left ischium. A finger introduced into the anus assured us that the intestine was not comprised in the laceration." (pp. 228.)

The treatment of the case was almost left to nature, and in five weeks the patient was perfectly cured. She became subsequently pregnant, and was safely delivered, with "only a slight laceration of the fourchette."

Thus we conclude our notice of M. Moreau's work. If it should be observed, that we have selected more passages and opinions to differ from than to praise, we answer that such has been our object; but in looking at the work as a whole we are inclined to speak of it in the highest terms, as one well adapted for the instruction of its readers and as equally well supporting even the high reputation so deservedly enjoyed by its author; and, without meaning to say that the pleasing appearance of a book is as good a letter of introduction as that of man or woman, we still think that a volume like this, so handsomely "got up" and splendidly and very plentifully illustrated, has attractions that most determined readers will properly appreciate and feel the comfort of, and that even their zest for its study is increased by good print and good paper. The translator appears to have well executed his task. The text reads smoothly and more unlike a translation than most transferences from one language to another.

